# Incidence of new-onset atrial fibrillation following phenylephrine vs. norepinephrine use in septic shock patients: A retrospective cohort study

**DOI:** 10.5339/qmj.2026.50

**Published:** 2026-08-11

**Authors:** Laith Rhabneh, Ra’ed Ababneh, Ibrahim Dib, Qusai Alqudah, Abdel Rahman Alwardat, Mohammed Aloqaily

**Affiliations:** 1 Internal Medicine Department, HMH Ocean University Medical Center, Brick, New Jersey, United States of America; 2 Anesthesia Department, Hamad Medical Corporation, Doha, Qatar; 3 Internal Medicine Department, HCA Florida North Florida Hospital, Gainesville, Florida, United States of America; 4 Internal Medicine Department, King Abdullah University Hospital, Irbid, Jordan; 5 Internal Medicine Department, University of Maryland Midtown Campus, Baltimore, Maryland, United States of America

**Keywords:** Atrial fibrillation, septic shock, vasopressors, norepinephrine, phenylephrine, critical care

## Abstract

**Background:**

Septic shock is a severe form of sepsis characterized by circulatory failure and organ dysfunction, often requiring vasopressor support to maintain adequate mean arterial pressure. Norepinephrine is the recommended first-line vasopressor, but new-onset atrial fibrillation (AF) is a common complication in septic shock patients receiving vasopressors, potentially influenced by vasopressor choice. This study compares the incidence of new-onset AF and related cardiac outcomes in septic shock patients treated with norepinephrine versus phenylephrine.

**Objective:**

To compare the incidence of new-onset AF within 72 hours of vasopressor initiation in adult septic shock patients treated with norepinephrine versus phenylephrine.

**Methods:**

A retrospective observational cohort study was conducted using the TriNetX database, including adult septic shock patients without prior AF who received either norepinephrine or phenylephrine. Propensity score matching balanced baseline characteristics across cohorts. The primary outcome was incidence of new-onset AF; secondary outcomes included cardioversion events, rate control medication use, other arrhythmias, and embolic stroke incidence. Outcomes were analyzed using Cox proportional hazards models and Kaplan–Meier survival curves.

**Results:**

After matching, 138,051 patients were included in each group. New-onset AF occurred in 0.3% of norepinephrine-treated patients versus 0.01% in the phenylephrine group (OR 3.05; 95% CI: 2.55–3.65; *p* < 0.001). Norepinephrine use was also associated with significantly increased rates of electrical and pharmacological cardioversion, rate control medication utilization, other arrhythmias, and embolic stroke (all *p* < 0.001). Kaplan–Meier analysis confirmed higher AF incidence in the norepinephrine group (log-rank *p* < 0.001).

**Conclusion:**

In septic shock patients, norepinephrine is associated with a significantly higher risk of new-onset AF and related adverse cardiac events compared to phenylephrine. These findings suggest vasopressor choice may influence arrhythmia risk and highlight the need for prospective studies to guide optimal vasopressor selection and management strategies in this population.

## 1. INTRODUCTION

Sepsis is a life-threatening organ dysfunction caused by an unregulated response of a host to infection. Septic shock is its most severe form, involving circulatory failure and profound organ impairment. Despite advancements in critical care, sepsis remains a leading cause of mortality among intensive care unit (ICU) patients.^1^ According to current guidelines, septic shock should be managed by treating the underlying cause and providing hemodynamic support to maintain a mean arterial pressure (MAP) of more than 65 mmHg. Norepinephrine is recommended as the first-line vasopressor for achieving this target.^2^

New-onset atrial fibrillation (AF) is a common complication in septic shock patients receiving vasopressors, with reported incidence ranging between 23–44%.^3^ The cause of the new-onset AF in this context is multifactorial, attributed to hemodynamic instability, systemic inflammatory response, and the arrhythmogenic effect of vasopressors.^4^

This study aims to address the existing knowledge gap by comparing the incidence of new-onset AF within 72 hours of vasopressor initiation in this high-risk population of adult patients with septic shock treated with norepinephrine versus phenylephrine. We examine other secondary outcomes, including the incidence of other cardiac arrhythmias, the need for cardioversion (electrical and pharmacological), rate control agents’ utilization, and embolic stroke incidence.

## 2. MATERIAL AND METHODS

### 2.1. Data Source

This study was conducted using the TriNetX research network, a federated database providing access to electronic health records (EHRs) from 153 healthcare organizations (HCOs). The TriNetX platform aggregates de-identified patient data, ensuring compliance with the Health Insurance Portability and Accountability Act (HIPAA) de-identification standards (§164.514(a)). More details on the data source can be found in previous TriNetX studies.

### 2.2. Patient Population

We conducted a retrospective observational cohort study, including adult patients (≥18 years old) with septic shock, categorized based on the vasopressor they initiated on, either norepinephrine or phenylephrine. Cohort 1 (norepinephrine group) included patients with septic shock who were started on norepinephrine, while Cohort 2 (phenylephrine group) included patients with septic shock who were started on phenylephrine. AF diagnosis was identified using the International Classification of Diseases, tenth version (ICD-10) codes.

While ECG (electrocardiogram) confirmation was not available in the database, the use of ICD-10 diagnostic codes for AF has been shown in prior validation studies to demonstrate a high positive predictive value (PPV) ranging from 85–95%, and vasopressor initiation was confirmed using RxNorm codes for norepinephrine and phenylephrine prescriptions. Additional details on cohort identification and study window definitions, including the relevant ICD-10, RxNorm, and Current Procedural Terminology (CPT®) codes, are provided in the Supplementary Appendix.

### 2.3. Study Endpoints

The index event was defined as the first recorded administration of either norepinephrine or phenylephrine for patients with septic shock, identified using medication codes RxNorm 7512 (norepinephrine) and RxNorm 8163 (phenylephrine). Sepsis was identified based on ICD-10 codes including A41.x (sepsis due to various organisms), R65.2 (severe sepsis), and other related subcodes (Supplementary Appendix). Patients with sepsis, severe sepsis, or septic shock were initially identified using ICD-10 codes. To ensure inclusion of only septic shock cases, patients not receiving vasopressors were excluded from the analysis. Patients were assigned to cohorts based on the initial vasopressor received; those who received norepinephrine without phenylephrine were placed in the norepinephrine cohort, while those who received phenylephrine without norepinephrine were placed in the phenylephrine cohort. The index date, defined as the time of initial vasopressor administration, marked the beginning of the observation window for evaluating outcomes.

The primary outcome of interest was the incidence of new-onset AF after vasopressor initiation. Secondary outcomes included cardioversion events (both electrical and pharmacological using agents such as amiodarone and procainamide), utilization of rate control agents (including beta-blockers, calcium channel blockers, or digoxin), occurrence of embolic stroke and other arrhythmias (other atrial arrhythmias and ventricular arrhythmias). Electrical cardioversion was determined based on ICD-10-PCS (Procedure Coding System) procedure codes. Use of rhythm or rate control agents was also determined using RxNorm codes. The Supplementary Appendix elaborates on outcome definitions and ICD-10 codes. 

### 2.4. Statistical Analysis

Continuous variables are presented as mean ± standard deviation (SD), whereas categorical variables are presented as number (percentage), as appropriate. Baseline characteristics were compared between the norepinephrine and phenylephrine groups using independent samples Student’s t-tests for continuous variables and chi-square tests for categorical variables. To mitigate baseline differences between cohorts, 1:1 propensity score matching (PSM) was performed using greedy nearest neighbor matching with a caliper of 0.1 times the pooled SD of the linear propensity scores. Variables included in the matching process were age, sex, race, body mass index (BMI), and comorbidities (hypertension, hyperlipidemia, diabetes mellitus, heart failure, other cardiac arrhythmias, paroxysmal tachycardia, atrioventricular and left bundle-branch block, ischemic heart disease, cerebral infarction, hypothyroidism, hyperthyroidism, chronic kidney disease [CKD], and tobacco use). The standardized mean difference represents the difference between the two groups in terms of SD units and is used to assess balance in measured variables in the sample weighted by the inverse probability of treatment. Variables were selected based on their potential impact on overall and cardiovascular outcomes.

After PSM, adjusted outcomes were compared between cohorts using hazard ratios (HRs) and 95% confidence intervals (CIs) derived from Cox proportional hazards regression models. Kaplan–Meier survival analysis was used to assess time-to-event outcomes, with differences between cohorts evaluated using the log-rank test. A p-value < 0.05 was considered statistically significant. All statistical analyses were conducted using integrated R (The R Foundation) within the TriNetX platform. TriNetX data undergo standardized quality assurance procedures, including automated error detection, deidentification checks, and routine validation by contributing institutions. Missing data for key variables were excluded listwise before propensity matching to ensure a complete-case analysis.

## 3. RESULTS

### 3.1. Study Population

**Figure 1.**
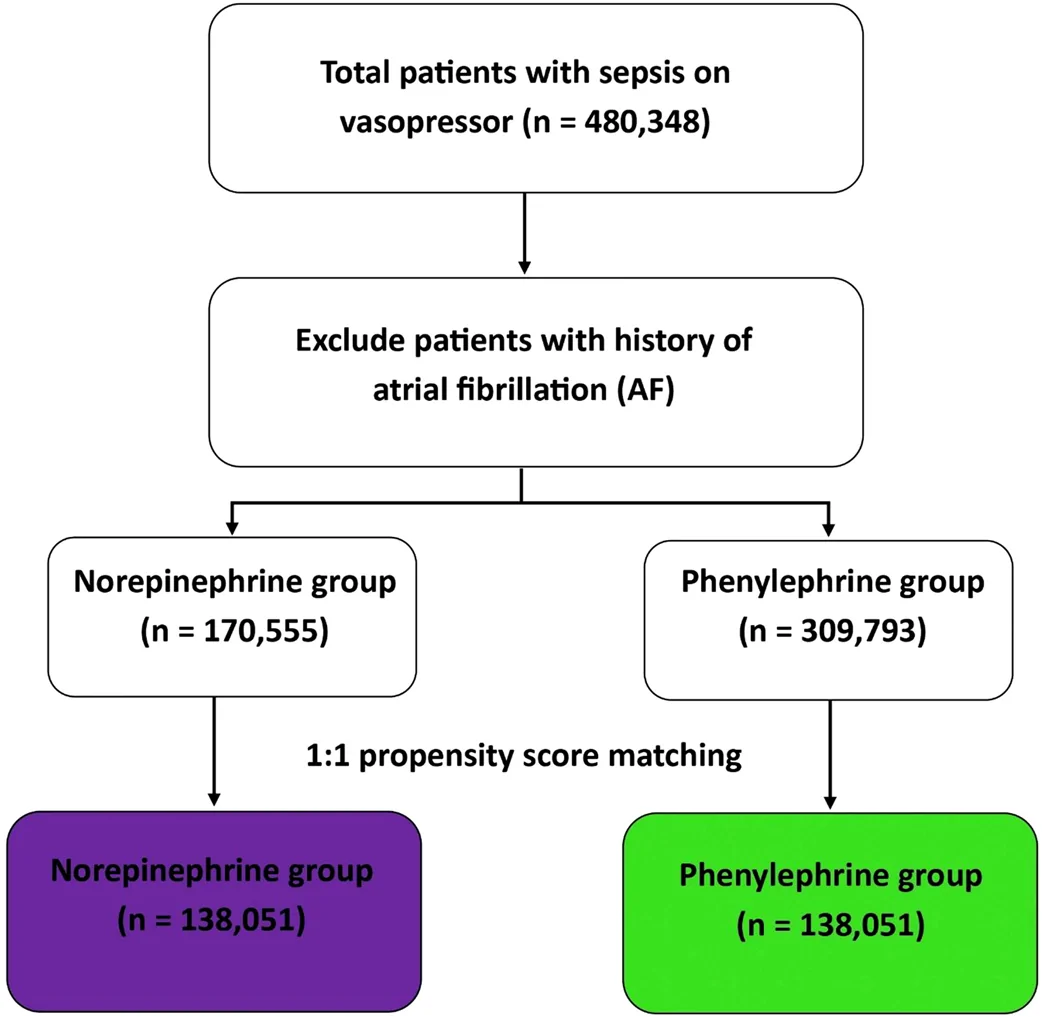
Flowchart of patient selection and propensity score matching (PSM) for sepsis cohort without pre-existing atrial fibrillation (AF).

This retrospective cohort study identified a total of 480,348 patients with a diagnosis of septic shock who were receiving vasopressors and had no prior history of AF. Among them, 170,555 patients received norepinephrine, and 309,793 patients received phenylephrine. After applying 1:1 PSM to balance baseline characteristics, 138,051 patients were included in each cohort (norepinephrine and phenylephrine groups) for the final analysis (Figure 1).

### 3.2. Patient Characteristics

The baseline characteristics of the study cohorts, before and after PSM, are shown in Table 1. In the unmatched cohort, patients receiving norepinephrine were slightly older at index (mean age: 62.4 ± 17.1 years vs. 58.2 ± 17.5 years, *p* < 0.001) compared to those receiving phenylephrine. The proportion of male patients was slightly higher in the norepinephrine group (53.1% vs. 48.3%, *p* < 0.001), while the phenylephrine group had a higher proportion of female patients (51.7% vs. 46.9%, *p* < 0.001). Regarding racial distribution, the phenylephrine group was more likely to be White (66.4% vs. 50.1%) compared to the norepinephrine group, while other racial differences were minor.

Before matching, the norepinephrine patient group exhibited a higher prevalence of cardiovascular risk factors, including heart failure (23.1% vs. 13.5%, *p* < 0.001), ischemic heart disease (29% vs. 23%, *p* < 0.001), and chronic kidney disease (24% vs. 21%, *p* < 0.001). Conversely, hypertension (63.1% vs. 53.3%, *p* < 0.001), diabetes mellitus (35.7% vs. 32.9%, *p* < 0.001), hyperlipidemia (45.9% vs. 33.5%, *p* < 0.001), tobacco use (7.1% vs. 4.7%, *p* < 0.001), overweight and obesity (28.9% vs. 19.2%, *p* < 0.001), and other cardiac arrhythmias (13.5% vs. 13.2%, *p* < 0.001) were more frequently observed in the phenylephrine patient group. Additionally, patients in the norepinephrine group had a higher prevalence of cerebrovascular disease, particularly cerebral infarction (8.7% vs. 6.8%, *p* < 0.001). The prevalence of thyroid disorders, including hypothyroidism and hyperthyroidism, was nearly the same between the two groups.

After PSM, the two cohorts were well balanced across key baseline characteristics, including age, sex, race, cardiovascular disease risk factors, and comorbidities, with standardized mean differences (SMDs) < 0.1 for most variables, indicating a well-matched cohort. The final matched cohort consisted of 138,051 patients in the norepinephrine group and 138,051 patients in the phenylephrine group. While sensitivity analyses, such as varying matching ratios or analyzing unmatched cohorts, were not performed in this study, TriNetX provides the capability to conduct such analyses. Future studies could leverage these approaches to further assess the robustness of observed associations.

### 3.3. Primary Outcome: Incidence of new-onset atrial fibrillation (AF)

**Figure 2.**
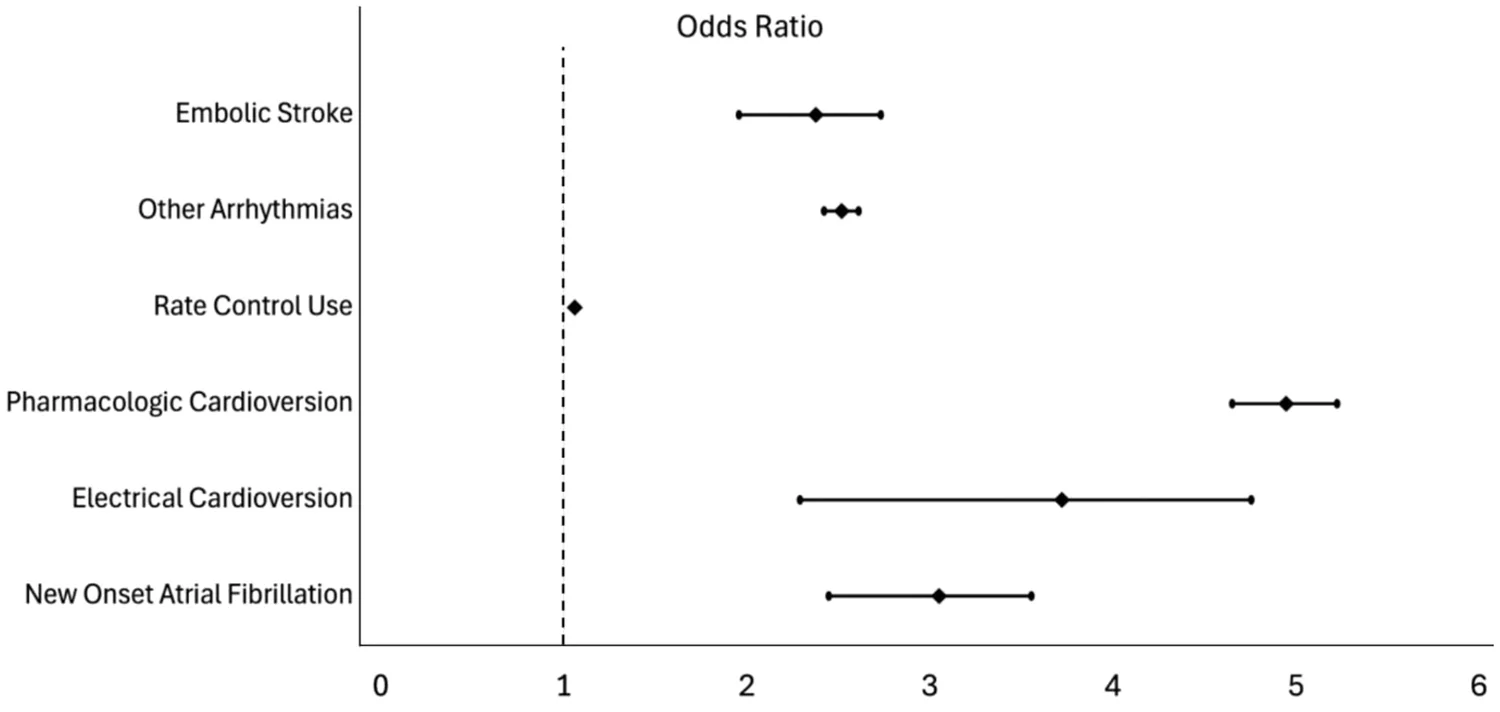
Forest plot of primary and secondary clinical outcomes: Norepinephrine vs. Phenylephrine in septic patients.

After matching, during the follow-up period, a total of 476 patients (0.3%) in the norepinephrine cohort experienced new-onset AF, compared to 157 patients (0.01%) in the phenylephrine cohort. Initiation of norepinephrine was associated with a significantly higher risk of new-onset AF (OR: 3.049; 95% CI: 2.545–3.652; *p* < 0.001). Kaplan–Meier survival analysis revealed a significantly higher incidence of new-onset AF in the norepinephrine group compared to the phenylephrine group (log-rank *p* < 0.001). The HR for developing AF was 3.055 (95% CI: 2.551–3.659), indicating that patients receiving norepinephrine had over three times the risk of developing new-onset AF relative to those receiving phenylephrine. The proportional hazards assumption was not violated (*p* = 0.545) (Table 2,Figure 2). Although the relative risk of AF was significantly higher in the norepinephrine group, the absolute difference in event rates was small (0.3% vs. 0.01%). This suggests that, while statistically significant, the clinical impact of this finding may be limited.

After matching, the secondary outcomes demonstrated significant differences between the norepinephrine group and the phenylephrine group. Electrical cardioversion events were more frequently reported in the norepinephrine group, with an odds ratio (OR) of 3.721 (95% CI: 2.687–5.153, *p* < 0.001), and an HR of 3.739 (95% CI: 2.700–5.177). Pharmacological cardioversion with amiodarone and procainamide was also more frequently reported in the norepinephrine group, with an OR of 4.945 (95% CI: 4.667–5.240, *p* < 0.001) and an HR of 4.858 (95% CI: 4.587–5.145). Rate control utilization was more commonly reported in the norepinephrine group, with an OR of 1.060 (95% CI: 1.028–1.093, *p* < 0.001) and an HR of 1.057 (95% CI: 1.026–1.088). A pooled analysis of cardioversion events demonstrated significantly higher odds in the norepinephrine group compared to the phenylephrine group (pooled OR: 4.92), combining both electrical and pharmacological cardioversion outcomes using inverse-variance weighted meta-analysis on the log scale. Other arrhythmia events were also more frequently reported in the norepinephrine group, with an OR of 2.517 (95% CI: 2.425–2.613, *p* < 0.001), and an HR of 2.432 (95% CI: 2.345–2.522). The risk of composite embolic stroke was significantly higher in the norepinephrine group compared to the phenylephrine group (OR: 2.376; 95% CI: 2.021–2.795, *p* < 0.001), and the HR was 2.381 (95% CI: 2.025–2.800) (Table 2Figure 2). Our study assessed multiple outcomes, including AF, cardioversion, rate control utilization, and stroke, without applying formal correction for multiple comparisons. As a result, there is an increased risk of type I error, and some statistically significant associations may have arisen by chance. Future studies should consider adjustment for multiplicity to validate these findings.

## 4. DISCUSSION

### 4.1. Arrhythmia Risk

In this retrospective cohort study of septic shock patients receiving vasopressors (norepinephrine or phenylephrine), we found that norepinephrine was associated with a significantly increased risk of new-onset AF compared to phenylephrine (OR: 3.049; 95% CI: 2.545–3.652; *p* < 0.001). We also observed a higher incidence of other cardiac arrhythmias in the norepinephrine group (OR: 2.517; 95% CI: 2.425–2.613; *p* < 0.001).

These findings are consistent with a retrospective study by Wieruszewski et al.^5^ which identified dysrhythmias in approximately one-third of septic shock patients treated with norepinephrine. On the other hand, not all data point toward harm. The Early Use of Norepinephrine in Septic Shock Resuscitation (CENSER) trial conducted by Permpikul et al.^6^ demonstrated a significantly lower incidence of new-onset arrhythmias in the early norepinephrine group compared to standard therapy (11% vs. 20%, *p* = 0.03).

Complementing these findings, a meta-analysis of randomized controlled trials by Ruslan et al.^7^ supports norepinephrine’s relatively favorable arrhythmia profile when compared to other vasopressors. The analysis found that norepinephrine was associated with a lower risk of arrhythmias (RR: 0.64; 95% CI: 0.42–0.97) across six trials involving nearly 4,000 patients. Nonetheless, it is important to note that phenylephrine was not consistently included in these comparisons.^7^

Taken together, these findings support the notion that β1-sparing agents like phenylephrine may be preferable in septic patients at increased risk for arrhythmias. In our cohort, the significantly elevated relative risk (RR ≈ 3.1) and consistently higher hazard over time lend weight to the hypothesis that norepinephrine’s β1-adrenergic activity contributes meaningfully to the development of AF in this population.

While our study reinforces this association, current guidelines and recommendations by the Infectious Diseases Society of America (IDSA) and the Society of Critical Care Medicine (SCC) continue to favor norepinephrine as first-line therapy in septic shock, with phenylephrine reserved for specific scenarios.^8^ Further research is needed to determine whether phenylephrine use translates into improved clinical outcomes, such as reduced AF-related complications. As this is an observational study, we can only establish associations rather than causality. The inability to determine the exact dose and duration of vasopressor use, as well as the lack of precise hemodynamic data, introduces potential confounding factors that may have influenced the observed outcomes.

### 4.2. Cardioversion

Restoration of normal rhythm in hemodynamically unstable patients is a critical intervention and should be achieved as promptly as possible, in accordance with Class I recommendations.^9^ Cardioversion, whether electrical or pharmacological, plays a central role in rhythm stabilization in such settings.

Norepinephrine, a catecholamine with both β_1_- and α_1_-adrenergic receptor activity, is known to exert arrhythmogenic effects by modulating cardiac ion channel activity and enhancing automaticity, particularly in atrial tissue.^10,11^ This mechanism likely contributes to the increased risk of arrhythmia observed in patients receiving norepinephrine.

In our study, patients in the norepinephrine group underwent significantly more cardioversion attempts compared to those receiving phenylephrine. The pooled OR for all cardioversion events was approximately 4.92. Pharmacological cardioversion was more frequently utilized, with an OR of 4.945 (95% CI: 4.667–5.240, *p* < 0.001), compared to electrical cardioversion, which had an OR of 3.721 (95% CI: 2.687–5.153, *p* < 0.001).

This preference for pharmacological over electrical cardioversion aligns with findings from prior studies, including the Control Atrial Fibrillation in Septic Shock (CAFS) study by Labbé et al.^12^ While data on cardioversion rates in this specific patient population remain limited, owing to the lack of large prospective studies or guideline-directed algorithms, current expert recommendations suggest initiating treatment with pharmacologic agents and reserving electrical cardioversion for patients who remain unstable or fail to respond to initial therapy.^9^

### 4.3. Rate control

Although the use of rate control medications in patients with septic shock receiving vasopressors is not routinely recommended and is not considered the standard of care, it may be considered on a case-by-case basis depending on the patient’s clinical condition.^13^ Despite limited discussion in the literature, a recent systematic review and meta-analysis by Al Sulaiman et al.^14^ reported that 5% to 30% of patients with septic shock on vasopressors received rate control medications. This is consistent with our study findings, where approximately 9% of patients received rate control medications. Notably, the rate was slightly higher in the norepinephrine group at 9.3%, compared to 8.8% in the phenylephrine group, which may be explained by the higher incidence of AF and other arrhythmias in the norepinephrine group.

### 4.4. Embolic stroke

To date, there is a notable gap in the literature regarding the association between vasopressor type and the risk of embolic stroke. Only one prior study by Polito et al.^15^ investigated the incidence of ischemic stroke in septic patients, some of whom received vasopressors. The study reported that 29% of patients developed ischemic stroke; however, it did not differentiate between embolic and thrombotic subtypes. Our study aimed to fill this gap by specifically evaluating the incidence of embolic stroke in patients treated with norepinephrine versus phenylephrine. Overall, the incidence of embolic strokes was approximately 0.3%, with a higher rate observed in the norepinephrine group (0.4%) compared to the phenylephrine group (0.2%). This difference may be partially explained by the higher incidence of new-onset AF in the norepinephrine group, a known independent risk factor for embolic stroke. This topic warrants further attention, and larger prospective studies are needed to explore this potential association and establish causality.

### 4.5. Limitations of the study

This study has several notable limitations. First, the data were sourced from the TriNetX EHR database, which relies on ICD codes to identify diagnoses and clinical characteristics. This approach is inherently susceptible to coding errors and biases common in real-world databases. Moreover, the database did not allow for the extraction of detailed medication information, such as dosage, adherence, or duration; we lacked precise data on timing, duration, and dose escalation, which may have influenced the results. Similarly, we lacked data on the duration and timing of vasopressor initiation—factors that may influence disease progression and outcomes. Although these limitations likely impacted both cohorts equally, our analysis could not quantify their potential effects on the observed outcomes.

Second, the study population was limited to patients with septic shock who were started on either norepinephrine or phenylephrine, which restricts the generalizability of our findings. Despite the use of PSM to minimize confounding bias, the possibility of residual confounding remains. Standardized illness severity scores such as Sequential Organ Failure Assessment (SOFA) or Acute Physiology and Chronic Health Evaluation II (APACHE II) were not available in the database and therefore could not be included in the analysis. This limitation may have introduced residual confounding related to unmeasured differences in disease severity between groups. Data on anticoagulation use, AF duration, and other key stroke risk factors were unavailable and could not be adjusted for in the analysis. As a result, the observed association with embolic stroke should be interpreted cautiously given potential residual confounding.

## 5. CONCLUSION

Our retrospective cohort analysis demonstrates that septic shock patients receiving norepinephrine experience significantly higher rates of new-onset AF, other cardiac arrhythmias, cardioversion (both electrical and pharmacological), rate control utilization, and embolic stroke compared to patients who received phenylephrine. Notably, we observed significantly more pharmacological cardioversion events than electrical ones. These findings highlight the differing outcomes associated with vasopressor selection in septic shock patients. Further prospective studies are needed to optimize treatment approaches, improve vasopressor selection strategies, and manage adverse outcomes in this high-risk population.

## ACKNOWLEDGEMENT

The author(s) have no acknowledgements to declare.

## AUTHORS’ CONTRIBUTIONS

LR: Conceptualization, Writing - Original Draft, Data Curation. RA: Writing - Review & Editing. ID: Writing - Original Draft. QA: Writing - Review & Editing, Data Curation. ARA: Writing - Review & Editing. MA: Writing - Review & Editing, Resources.

## CONFLICT OF INTEREST

The authors declare that they have no conflicts of interest related to this study.

## DISCLOSURE OF AI USE

No artificial intelligence tools were used in the preparation, writing, editing, or analysis of this manuscript.

## DATA AVAILABILITY

The data used in this study were obtained from the TriNetX platform. Due to the nature of the data and the terms of use of the TriNetX network, the underlying patient-level data are not publicly available. Qualified researchers may access the data through TriNetX in accordance with its data-use policies and applicable requirements.

## FUNDING STATEMENT

The authors confirm that no financial support was received for this study. This research did not receive any specific grant from funding agencies in the public, commercial, or not-for-profit sectors.

## ETHICAL APPROVAL

This study used de-identified data from the TriNetX research network. In accordance with U.S. federal regulations, studies using exclusively de-identified data are not considered human subjects research and are therefore exempt from institutional review board (IRB) approval.

## DECLARATION OF PATIENT CONSENT

Not applicable. The study used anonymized data only and did not involve identifiable individual participants.

## SUPPLEMENTARY APPENDIX

**Appendix. ICD-10, RxNorm, and CPT codes used to define the study cohorts, exposure groups, exclusion criteria, and clinical outcomes.**



## Figures and Tables

**Table 1.** Baseline characteristics of patients in norepinephrine and phenylephrine groups before and after propensity score matching (PSM).

	Before PSM			After PSM		
	Before matching (Norepinephrine group, *n* = 170,555)	Before matching (Phenylephrine group, *n* = 309,793)	Standardized difference	After matching (Norepinephrine group, *n* = 138,051)	After matching (Phenylephrine group, *n* = 138,051)	Standardized difference
**Demographics**						
Current age (Mean ± SD)	67.5 ± 16.7	64.0 ± 17.4	0.207	65.9 ± 16.7	65.9 ± 17.2	<0.001
Age at Index (Mean ± SD)	62.4 ± 17.1	58.2 ± 17.5	0.239	60.5 ±- 17.0	60.6 ±17.4	0.005
Female (%)	79,631 (46.9%)	159,138 (51.7%)	0.095	66,969 (48.5%)	67,080 (48.6%)	0.002
Male (%)	90,088 (53.1%)	148,847 (48.3%)	0.095	71,082 (51.5%)	70,971 (51.4%)	0.002
White (%)	85,058 (50.1%)	204,552 (66.4%)	0.335	81,634 (59.1%)	80,706 (58.5%)	0.014
**Comorbid conditions**						
Hypertension	90,500 (53.3%)	194,282 (63.1%)	0.199	79,831 (57.8%)	80,884 (58.6%)	0.015
Heart failure	39,210(23.1%)	41,530 (13.5%)	0.251	29,677 (21.5%)	28,854 (20.9%)	0.015
Other cardiac arrhythmias	22,452 (13.2%)	41,431 (13.5%)	0.007	19,590 (14.2%)	19,255 (13.9%)	0.007
Ischemic heart diseases	49,146 (29.0%)	70,776 (23.0%)	0.137	39,544 (28.6%)	39,270 (28.4%)	0.004
Cerebral infarction	14,684 (8.7%)	20,926 (6.8%)	0.070	11,496 (8.3%)	11,331 (8.2%)	0.004
Diabetes mellitus	55,856 (32.9%)	109,797 (35.7%)	0.058	47,570 (34.5%)	48,243 (34.9%)	0.010
Hypothyroidism	20,330 (12.0%)	44,066 (14.3%)	0.069	18,543 (13.4%)	18,599 (13.5%)	0.001
Hyperthyroidism	2,317 (1.4%)	5,514 (1.8%)	0.034	2,075 (1.5%)	2,030 (1.5%)	0.003
Chronic kidney disease	40,656 (24.0%)	64,574 (21.0%)	0.072	32,922 (23.8%)	32,769 (23.7%)	0.003
Overweight and obesity	32,611 (19.2%)	88,882 (28.9%)	0.227	31,198 (22.6%)	31,695 (23.0%)	0.009
Hyperlipidemia	56,805 (33.5%)	141,475(45.9%)	0.257	52,649 (38.1%)	54,074 (39.2%)	0.021
Tobacco use	7,946 (4.7%)	21,902 (7.1%)	0.103	7,614 (5.5%)	7,501 (5.4%)	0.004

**Table 2.** Primary and secondary clinical outcomes: Norepinephrine vs. Phenylephrine in septic patients.

Outcome	Risk of event (Norepinephrine group)	Risk of event (Phenylephrine group)	Odds ratio (95% CI)	*p*-value
**Primary outcome**				
New-onset atrial fibrillation (AF)	476 (0.3%)	157 (0.01%)	3.049 (2.545–3.652)	< 0.001
**Secondary outcome**				
Electrical cardioversion	171 (0.12%)	46 (0.03%)	3.721 (2.687–5.153)	< 0.001
Pharmacological cardioversion	6,647 (4.9%)	1,414 (1%)	4.945 (4.667–5.240)	< 0.001
Rate control	9,287 (9.3%)	8,462 (8.8%)	1.060 (1.028–1.093)	< 0.001
Other arrhythmias	10,168 (9%)	4,118 (3.8%)	2.517 (2.425–2.613)	< 0.001
Embolic stroke	493 (0.4%)	208 (0.2%)	2.376 (2.021–2.795)	< 0.001
