# Supplementary material for: Incidence of new-onset atrial fibrillation following phenylephrine vs. norepinephrine use in septic shock patients: A retrospective cohort study
Source: Qatar Med J. 2026 Aug 11;2026(3):50. doi: 10.5339/qmj.2026.50 (PMC13547784; doi:10.5339/qmj.2026.50)
Supplement: Supplementary file 1 [file qmj-2026-03-050.supp1.docx]

**Appendix. ICD-10, RxNorm, and CPT codes used to define the study cohorts, exposure groups, exclusion criteria, and clinical outcomes.**

| **Category** | **Code System** | **Code** | **Description** | **Purpose** |
| --- | --- | --- | --- | --- |
| **Sepsis Cohort** | ICD-10 | A41.9 | Sepsis, unspecified organism | Inclusion |
|  | ICD-10 | R65.2 | Severe sepsis | Inclusion |
|  | ICD-10 | R65.20 | Severe sepsis without septic shock | Inclusion |
|  | ICD-10 | R65.21 | Severe sepsis with septic shock | Inclusion |
|  | ICD-10 | A41.89 | Other specified sepsis | Inclusion |
|  | ICD-10 | A41.0 | Sepsis due to *Staphylococcus aureus* | Inclusion |
|  | ICD-10 | A41.1 | Sepsis due to other specified staphylococcus | Inclusion |
|  | ICD-10 | A41.2 | Sepsis due to unspecified staphylococcus | Inclusion |
|  | ICD-10 | A41.3 | Sepsis due to *Haemophilus influenzae* | Inclusion |
|  | ICD-10 | A41.4 | Sepsis due to anaerobes | Inclusion |
|  | ICD-10 | A41.50 | Gram-negative sepsis, unspecified | Inclusion |
|  | ICD-10 | A41.51 | Sepsis due to *Escherichia coli* | Inclusion |
|  | ICD-10 | A41.52 | Sepsis due to *Pseudomonas* | Inclusion |
|  | ICD-10 | A41.59 | Other Gram-negative sepsis | Inclusion |
| **Exposure** | RxNorm | 7512 | Norepinephrine | Cohort 1 |
|  | RxNorm | 8163 | Phenylephrine | Cohort 2 |
| **Baseline AF Exclusion / Outcome** | ICD-10 | I48 | Atrial fibrillation and flutter | Outcome |
|  | ICD-10 | I48.0 | Paroxysmal atrial fibrillation | Exclusion/Outcome |
|  | ICD-10 | I48.1 | Persistent atrial fibrillation | Exclusion/Outcome |
|  | ICD-10 | I48.11 | Longstanding persistent atrial fibrillation | Exclusion/Outcome |
|  | ICD-10 | I48.19 | Other persistent atrial fibrillation | Exclusion/Outcome |
|  | ICD-10 | I48.2 | Chronic atrial fibrillation | Exclusion/Outcome |
|  | ICD-10 | I48.20 | Chronic atrial fibrillation, unspecified | Exclusion/Outcome |
|  | ICD-10 | I48.21 | Permanent atrial fibrillation | Exclusion/Outcome |
|  | ICD-10 | I48.9 | Unspecified atrial fibrillation and atrial flutter | Outcome |
|  | ICD-10 | I48.91 | Unspecified atrial fibrillation | Exclusion/Outcome |
| **Electrical Cardioversion** | CPT | 1012978 | Cardioversion, elective, electrical conversion of arrhythmia | Outcome |
| **Embolic Stroke** | ICD-10 | I63.4 | Cerebral infarction due to embolism of cerebral arteries | Outcome |
| **Other Arrhythmias** | ICD-10 | R00.0 | Tachycardia, unspecified | Outcome |
|  | ICD-10 | I47 | Paroxysmal tachycardia | Outcome |
|  | ICD-10 | I47.1 | Supraventricular tachycardia | Outcome |
|  | ICD-10 | I47.10 | Supraventricular tachycardia, unspecified | Outcome |
|  | ICD-10 | I47.11 | Inappropriate sinus tachycardia | Outcome |
|  | ICD-10 | I47.19 | Other supraventricular tachycardia | Outcome |
|  | ICD-10 | I47.2 | Ventricular tachycardia | Outcome |
|  | ICD-10 | I47.20 | Ventricular tachycardia, unspecified | Outcome |
|  | ICD-10 | I47.29 | Other ventricular tachycardia | Outcome |
|  | ICD-10 | I47.9 | Paroxysmal tachycardia, unspecified | Outcome |
|  | ICD-10 | I49.0 | Ventricular fibrillation and flutter | Outcome |
|  | ICD-10 | I49.01 | Ventricular fibrillation | Outcome |
|  | ICD-10 | I49.02 | Ventricular flutter | Outcome |
| **Rate Control Agents** | RxNorm | 6918 | Metoprolol | Outcome |
|  | RxNorm | 3443 | Diltiazem | Outcome |
|  | RxNorm | 3407 | Digoxin | Outcome |
|  | RxNorm | 20352 | Carvedilol | Outcome |
|  | RxNorm | 11170 | Verapamil | Outcome |
|  | RxNorm | 9947 | Sotalol | Outcome |
| **Pharmacologic Cardioversion** | RxNorm | 703 | Amiodarone | Outcome |
|  | RxNorm | 8700 | Procainamide | Outcome |
